# Return to sports after unilateral medial opening wedge high tibial osteotomy in highly active patients: Analysis of factors affecting functional recovery

**DOI:** 10.1002/jeo2.70083

**Published:** 2025-01-03

**Authors:** Hiroshi Nakayama, Ryo Kanto, Shintaro Onishi, Takuya Iseki, Yoshitaka Nakao, Toshiya Tachibana, Kenta Amai, Shinichi Yoshiya, Tomoya Iseki

**Affiliations:** ^1^ Department of Orthopaedic Surgery Hyogo Medical University Hyogo Japan; ^2^ Department of Orthopaedic Surgery Nishinomiya Kaisei Hospital Hyogo Japan; ^3^ Department of Orthopaedic Surgery Osaka Kaisei Hospital Osaka Japan

**Keywords:** athletes, high tibial osteotomy, knee, osteotomy, return to sports, sports

## Abstract

**Purpose:**

The purpose of this study was to examine the outcomes following opening‐wedge high tibial osteotomy (HTO) focusing on return to sports in a consecutive series of highly active patients who underwent a unilateral osteotomy procedure.

**Methods:**

Sixty‐three consecutive patients with preoperative Tegner's activity score of five or more who underwent unilateral HTO for varus osteoarthritic knees were included in this study. The clinical results were evaluated using the Knee Injury and Osteoarthritis Outcome Score (KOOS) and the International Knee Documentation Committee (IKDC) Subjective Score. In radiological assessment, the following parameters were measured in full‐length weight‐bearing radiographs both pre‐ and postoperatively; mechanical tibiofemoral angle (mTFA), mechanical medial proximal tibial angle (mMPTA) and joint‐line convergence angle. As regard postoperative functional recovery, inability to return to sports activities and reduction in the activity level on the Tegner scale were considered as failure to return to sports. Potential prognostic factors examined with logistic regression analysis were as follows: age ≥ 70, body mass index > 25, postoperative mTFA > 3° valgus or <0° varus, postoperative mMPTA > 90°, opening gap > 10 mm and Kellgren–Laurence classification (KL) grade 4.

**Results:**

At 2 years after surgery, the KOOS and the IKDC score improved from 231 to 437 and from 34 to 72, respectively, with significant improvements in both scores. As for functional recovery, 50 patients (79.4%) could return to high‐impact sports activities at the presymptomatic level with a mean time period of 8.0 months. Statistical analysis of the prognostic factors showed that postoperative mTFA > 3° valgus, opening gap >10 mm and KL grade 4 were the factors significantly affecting the postoperative return to sports.

**Conclusions:**

Presence of postoperative mTFA > 3° valgus, opening gap >10 mm and KL grade 4 were identified as risk factors impairing postoperative return to high‐impact sports.

**Level of Evidence:**

Level Ⅳ.

**Abbreviations**
BMI: body mass index
HTO: high tibial osteotomy
IKDC: the International Knee Documentation Committee
JLCA: joint‐line convergence angle
KL: Kellgren–Laurence
KOA: knee osteoarthritis
KOOS: the Knee Injury and Osteoarthritis Outcome Score
mMPTA: mechanical medial proximal tibial angle
mTFA: mechanical tibiofemoral angle
OWDTO: opening‐wedge distal tuberosity osteotomy
OWHTO: opening‐wedge high tibial osteotomy
TKA: total knee arthroplasty
WBL: weight‐bearing line

## INTRODUCTION

Osteotomy around the knee is well‐established and a commonly employed surgical option for active patients with unicompartmental knee osteoarthritis (KOA) [6, 12, 13]. High tibial osteotomy (HTO) offers several advantages over total knee arthroplasty (TKA) in younger patients, including preservation of the native joint and an absence of permanent activity restrictions in contrast to TKA, in which activity modification may be required to achieve durability of the prosthesis [4]. In accordance with evolution of surgical technique and instruments, indication of HTO has been expanded to those who are relatively young and active. Consequently, the aim of surgery in the patient population has been shifted from improvement in daily living activities to return to sports and/or work, and also inability to return after surgery can negatively impact the quality of life and patient's satisfaction for surgery [7].

There have been several studies investigating return to sports following HTO; [3, 8, 13, 18, 19] however, reported rates of return to sports are varied among the studies and efficacy of HTO in functional recovery for athletes has not been clarified. The inconsistent results in the previous relevant studies can be attributed to the shortcomings included in the study design as follows. First, most of those reports included low‐activity patients participating in low‐impact sports (e.g., hiking, walking, cycling, swimming). Second, patients undergoing bilateral HTOs were included, which impairs accurate assessment of isolated effect of surgery and time to return to sports. Third, the level of sports activities attained after surgery was not well defined. In addition, surgical procedure and postoperative rehabilitation are varied among the studies. In order to address those problems, in the present study, study subjects were limited to highly active patients who participated in high‐impact sports before surgery with the presymptomatic Tegner activity level of ≥5 and underwent unilateral HTO procedure.

The purpose of this study was to examine the outcomes following opening‐wedge HTO (OWHTO) or opening‐wedge distal tuberosity osteotomy (OWDTO) focusing on return to sports in a consecutive series of highly active patients who underwent a unilateral osteotomy procedure desiring to resume their preoperative sports activity level. It was hypothesized that these osteotomies would achieve high rate of return to high‐impact sports, and there would be some prognostic factors affecting postoperative functional recovery.

## METHODS

### Patient population

**Figure 1:**
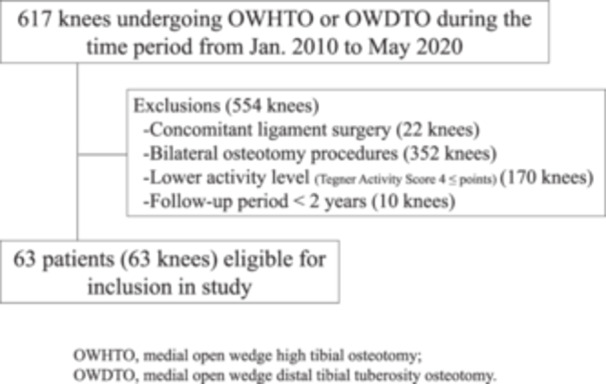
Flowchart for patient selection. OWDTO, medial open wedge distal tibial tuberosity osteotomy; OWHTO, medial open wedge high tibial osteotomy.

This is a single‐center retrospective study. As a study population, consecutive patients with KOA who underwent medial OWHTO [17, 27] between April 2010 and July 2018 and those who underwent medial open‐wedge distal tibial tuberosity osteotomy (OWDTO) [2] from August 2018 to May 2020 were initially enrolled in the study. The OWDTO procedure is a modification of OWHTO in which anterior vertical osteotomy extending distal to the tibial tubercle is added to the transverse OWHTO. In our practice, the osteotomy procedure for correction of varus deformity at the proximal tibia has been changed from OWHTO to OWDTO in August 2018 with the intent of avoiding postoperative patella infra. Our indications for OWHTO or OWDTO were (1) symptomatic medial KOA or necrosis with varus malalignment, (2) range of motion of 120° or more of flexion and <10° of extension loss and (3) absence of advanced patellofemoral OA (Kellgren and Lawrence (KL) grade 3 or more). Of the 617 knees in 436 patients who were initially enrolled, those who participated in sporting activities corresponding to the Tegner activity score of ≥5 during presymptomatic period [29] and opted to receive the osteotomy desiring to resume high‐level sports activity were included in the study. Thereafter, patients meeting the following exclusion criteria were excluded: (1) bilateral HTO procedures, (2) concomitant reconstructive surgeries for ligament insufficiency and (3) lack of clinical/radiological data at 2 years (Figure 1). All cases underwent approximately 3 months of conservative treatment without success, and due to the strong desire to return to sports, HTO was performed.

### Clinical and radiological evaluations

In evaluation of overall clinical outcome, validated patient‐registered outcome measures, the Knee Injury and Osteoarthritis Outcome Score (KOOS) and the International Knee Documentation Committee (IKDC) Subjective Knee Score were used for quantitative evaluation before surgery and at the follow‐ups. As for radiological evaluations, the following parameters were measured using the dedicated software (mediCAD) [26] on a long‐leg weight‐bearing radiograph: mechanical medial proximal tibial angle (mMPTA), joint‐line convergence angle (JLCA), mechanical tibiofemoral angle (mTFA) and weight‐bearing line (WBL) ratio. The WBL ratio is determined by the intersection point of the mechanical axis and the tibial plateau (with the medial and lateral tibial edges corresponding to 0 and 100%). The clinical and radiological evaluations were conducted preoperatively and at the follow‐ups.

### Assessment of return to sports

In assessment of return to sports, the type of sports, the time to return to the sporting activity and the return rate were examined. The Tegner activity scale was used to determine activity levels. The status of sports activity was assessed both before and after surgery. Before surgery, most of the patients were forced to reduce their activity level due to pain. Therefore, the preoperative level was determined as the level before the onset of the knee symptoms, while postoperative sports activity level was assessed for the highest level attained after surgery. Criteria for failure to return to sports were as follows: (1) inability to return to sports at the presymptomatic level or (2) postoperative reduction in activity level on the Tegner activity scale. As for the factors that potentially affect the postoperative functional recovery, the following factors were subjected to the analyses: patients' age ≥ 70 years at surgery, body mass index > 25, postoperative mTFA > 3°, postoperative mTFA < 0°, postoperative mMPTA > 90°, opening gap > 10 mm and KL grade 4 [15].

### Surgical procedure

All surgeries were performed by the first author (H. N.). General anesthesia was used for all patients. Arthroscopy was performed before the osteotomy to evaluate the intraarticular pathologies and arthroscopic procedures such as meniscal surgery was conducted as needed.

The OWHTO was performed via a modification of the technique which was previously reported by Staubli and Lobenhoffer [17, 27]. A bone substitute (ß‐TCP: Osferion 60; Olympus Terumo Biomaterials) was placed in the osteotomy gap, while fixation was achieved using a TomoFix TM medial high tibial plate (DePuy Synthes). In August 2018, OWDTO was induced to our practice with the intent of preventing patellar infra caused by wedge opening proximally to the tibial tubercle [2]. Since then, OWDTO using the TriS plate (Olympus) has been our primary option in proximal tibial osteotomy.

### Postoperative rehabilitation and follow‐up evaluations

The operated knee was not immobilized after surgery. Range of motion exercise was started at 1‐day without any limitations. Partial weight‐bearing with 20 kg was allowed at 3 weeks using crutches with progression to full weight‐bearing at 4 weeks in OWHTO and 6 weeks in OWDTO. In patients with concomitant autologous osteochondral transplantation, active free range of motion and weight‐bearing were not allowed for 4 weeks. At 3 months postoperatively, patients were permitted to resume jogging provided that bone healing at the osteotomy site was attained on radiographs. Finally, patients were allowed to return to sports activities after 6 months following confirmation of adequate functional recovery including muscle strength and complete bony healing. Periodical postoperative follow‐up evaluations were performed every 3 months within a year and then every 6 months afterward. Comprehensive radiological and clinical evaluations were conducted at 12 and 24 months after surgery, and the obtained results were compared to the preoperative status.

The study design was approved by the Institutional Review Board of Hyogo Medical University [No. 2218], and informed consent was obtained from all study subjects.

### Statistical analysis

To assess the statistical power of this study, a post hoc power analysis was conducted for comparison of the pre‐ and postoperative clinical KOOS scores using a two‐sided test. Consequently, it was shown that the total sample size of 63 in this study could achieve an adequate power 1 − *β* of 0.81 with an *α* of 0.05, using free statistical power analysis software (G*Power, version 3.1.9.2; Franz Faul, Universitat Kiel).

In statistical analysis, the Mann–Whitney's U test was used for comparisons of pre‐ versus postoperative clinical scores and radiological parameters, as well as comparison of patient's factors between the groups of patients with successful return to sports and those with failure to attain satisfactory functional recovery. Univariate analysis of potential risk factors was performed using the Fisher's exact test. Factors found to have values of *p* < 0.1 in the univariate analysis were included in the subsequent multivariate logistic regression analysis. Results are summarized as odds ratios, 95% confidence intervals and *p* values. All *p* values were two‐sided, and *p* < 0.05 was considered statistically significant. Statistical analyses were performed using SPSS (version 19, SPSS Inc.).

## RESULTS

### Patient selection and profiles

As shown in Figure 1, 554 patients of the 617 initially enrolled patients were excluded from the study based on the exclusion criteria, leaving 63 patients (63 knees) as a study population subjected to the analysis. The demographics and clinical profiles of these patients are presented in Table 1. There were 44 males and 19 females with a mean age of 55.8 ± 10.1 years (range, 31–75 years). All patients were followed‐up for at least 2 years after surgery, and the mean time between the surgery and the follow‐up of the study group was 61.4 ± 20.3 months (range, 24–102 months). According to Ahmad et al., a minimum mean orthopedic follow‐up of a short‐term study should be 30 months [1]. In our study, five of the 63 patients (7.9%) had at least 30 months of follow‐up.

**Table 1:** Demographics and clinical profile of the study population.

Total patients, *n*	63
Total knees, *n*	63
OWHTO, *n*	45
OWDTO, *n*	18
Sex	Male 44/Female 19
Side	Right 34/Left 29
Age at surgery (years)	55.8 ± 10.1^a^ (range, 1–75)
BMI (kg/m^2^)	24.3 ± 2.8^a^ (range, 18.1–31.0)
Mean time between the surgery and the follow‐up (months)	61.4 ± 20.3^a^ (range, 24–102)
Concomitant procedures during osteotomy	
Any, *n* (%)	40 (63.5%)
Meniscal, *n*	33
Debridement	23
Repair	10
Chondral, *n*	9
Microfracture	6
Osteochondral autograft transfer	3
Wedge size (mm)	7.9 ± 2.9^a^ (range, 4–15)
Implant removal, *n* (%)	63 (100%)
Mean time between osteotomy and hardware removal (months)	11.5 ± 3.9^a^ (range, 5–31)
Presymptomatic sports activity level based on the Tegner's scale	5.3 ± 0.6^a^ (range, 5–7)

Abbreviations: BMI, body mass index; OWDTO, medial open‐wedge distal tibial tuberosity osteotomy; OWHTO, medial open‐wedge high tibial osteotomy.

^a^ Values are expressed as mean ± standard deviation.

Concomitant procedures were performed in 40 patients (63.5%), including 33 meniscal and nine chondral procedures (including two knees with combined meniscal/chondral procedures). All patients (100%) underwent hardware removal at 11.5 ± 3.9 months on average after the index surgery (Table 1).

### Clinical results

In terms of clinical outcomes, the detailed results of the clinical scores (IKDC subjective score and KOOS) are shown in Table 2. Compared to preoperative scores, the IKDC subjective score, as well as KOOS in all of the five subscales exhibited significant improvements (*p* < 0.05) at 12 and 24 months postoperatively.

**Table 2:** Clinical scores based on patient‐reported outcome measures.

Scores	*n* = 63 knees		
	Preoperative	12 months after surgery	24 months after surgery
IKDC (points)	34 ± 14.3	67 ± 11.1^a^	72 ± 9.5^a^
KOOS (points)			
Symptom	50.2 ± 20.4	84.7 ± 13.8^a^	87.0 ± 12.0^a^
Pain	48.4 ± 19.5	89.4 ± 10.9^a^	90.9 ± 9.5^a^
ADL	65.7 ± 18.6	94.9 ± 6.8^a^	96.0 ± 6.0^a^
Sports	31.9 ± 22.5	80.0 ± 15.3^a^	84.0 ± 15.1^a^
QOL	35.0 ± 19.5	77.4 ± 19.1^a^	80.0 ± 15.5^a^
Total	231.4 ± 85.5	426.8 ± 55.6^a^	437.8 ± 48.3^a^

*Note*: Values are expressed as mean ± standard deviation.

Abbreviations: ADL, activities of daily living; IKDC, International Knee Documentation Committee; KOOS, Knee Injury and Osteoarthritis Outcome Score; QOL, quality of life.

^a^ Statistically significant improvement compared to the preoperative score (*p* < 0.05).

As for postoperative complications, a surgical site infection occurred in one patient (1.6%) who was a 57‐year‐old male with a body mass index of 27.9 kg/m^2^. Debridement without removal of the plate combined with antibiotic administration was performed. The infection had subsided leading to successful return to sports at 10 months after the primary surgery. No other osteotomy‐related complications were found. Hardware removal has been routinely performed for all patients including nonsymptomatic patients, and thus was not counted as a revision surgery.

### Radiological results

Preoperatively, all knees exhibited varus deformity with the mean mTFA angle of 5.4° ± 2.5° varus (range, 0°–12.0° varus). The mean mMPTA and the mean JLCA were 84.7 ± 2.0° (range, 78.0°–87.0°) and 2.4 ± 2.0°, respectively. The mean WBL ratio was 26.0 ± 10.3% (range, −13% to 47.6%). Preoperative KL grade among the study patients were as follows: grade 1, four knees (6.3%); grade 2, 25 knees (39.7%); grade 3, 29 knees (46.0%) and grade 4, five knees (7.9%). The Radiological evaluation at 24 months showed restoration of normal knee alignment with the mean mTFA significantly corrected to 1.7° ± 2.2 valgus (range, –1.1° to 4.0° valgus) and the WBL ratio (medial border is 0%, lateral border is 100%) corrected to 53.5 ± 2.5% (range, 46%–62.4%). The mean mMPTA and the JLCA were 90.7 ± 2.6° and 1.7 ± 1.4°, respectively. There was no progression in KL grade in femorotibial joint during the follow‐up period (Table 3).

**Table 3:** Pre‐ and postoperative radiological parameter values of alignment and orientation of the knee.

	Preoperative	2‐year after surgery
mTFA	5.4 ± 2.5° varus	1.7 ± 2.2°valgus
mMPTA	84.7 ± 2.0°	90.7 ± 2.6°
JLCA	2.4 ± 2.0°	1.7 ± 1.4°
%MA	26.0 ± 10.3%	53.5 ± 2.5%

*Note*: Values are expressed as mean ± standard deviation.

Abbreviations: %MA, % mechanical axis; JLCA, joint‐line convergence angle; mMPTA, mechanical medial proximal angle; mTFA, mechanical tibiofemoral angle.

### Return to sports

Out of 63 patients, 50 patients (79.4%) returned to high‐impact sports activities at the presymptomatic level after surgery with the mean time to return of 8.0 ± 2.4 months (range, 6–14 months). Detailed data for pre‐ and postoperative participations in each type of sports activity are presented in Table 4. In the 13 patients who failed to regain the presymptomatic sports activity level, seven patients (11.1%) returned to sports at reduced activity level while six patients (9.5%) did not return to sports after surgery. Regarding the reasons for stoppage of sports activity, two patients (3.2%) cited persistent pain and swelling, and four patients (6.3%) cited loss of motivation to play sports. As for the overall sports activity data on the Tegner activity scale, the activity level averaged 5.3 ± 0.6 preoperatively and significantly decreased to 5.0 ± 1.0 at 2 years after surgery (*p *< 0.05) (Table 4).

**Table 4:** Participation in sports activities in the study population before and after surgery.

Sports activities	Before surgery	After surgery	Rate of return to sports (%)
Jogging	36	27	75.0
Tennis	7	6	85.7
Baseball	3	2	66.7
Dance	3	2	66.7
Boxing	2	2	100
Volleyball	2	2	100
Marathon	2	1	50
Soccer	2	2	100
Triathlon	1	1	100
Judo	1	1	100
Self‐defense force	1	1	100
Bodybuilding	1	1	100
Basketball	1	1	100
Swimming	1	1	100
Total	63	50	79.4
Tegner's score	5.3 ± 0.6 (range, 5–7)	5.0 ± 1.0 (range, 2–7)	

In the analysis of risk factors for failure to regain original sports activity, the univariate analysis demonstrated the following factors as potential risk factors with a *p* < 0.05: postoperative mTFA > 3° valgus, opening gap > 10 mm and KL grade 4 (Table 5). In the subsequent multivariate logistic regression analysis, all of those three factors were identified as risk factors significantly affecting the postoperative functional recovery (Table 6).

**Table 5:** Univariate analysis of risk factors for poor prognosis using the Ficher's exact test.

Risk factor	*p* Value
Age ≥70 years	0.49
BMI > 25	0.42
Postoperative mTFA > 3°valgus	0.002
Postoperative mTFA < 0°	0.60
Postoperative mMPTA > 90°	0.30
Opening gap > 10 mm	0.002
KL grade 4	0.006

Abbreviations: BMI, body mass index; KL, Kellgren–Lawrence classification; mMPTA, mechanical medial proximal tibial angle; mTFA, mechanical tibiofemoral angle.

**Table 6:** Multivariate logistic regression analysis of risk factors for poor prognosis.

Risk factor	*p* Value	Odds ratio	95% CI
Postoperative mTFA > 3°valgus	0.003	15.0	2.4–90.9
Opening gap > 10 mm	0.003	9.9	2.2–43.9
KL grade 4	0.009	21.8	2.1–218.0

Abbreviations: CI, confidence interval; mTFA, mechanical tibiofemoral angle; KL, Kellgren–Lawrence classification.

## DISCUSSION

The most important finding of this study was that patients who underwent unilateral OWHTO or OWDTO returned to high‐impact sports at their presymptomatic level (Tegner activity scale ≥ 5) with a return rate of 79.4% (50 of the 63 patients) postoperatively with the mean time to return of 8.0 months. In the remaining 13 patients with less satisfactory functional recovery, seven patients (11.1%) returned to sports at reduced level and six patients (9.5%) stopped playing any sports including two patients due to knee symptoms. As for the factors affecting the postoperative functional recovery, postoperative valgus alignment of more than 3°, large opening gap of more than 10 mm and advanced osteoarthritis corresponding to KL grade 4 were identified as factors significantly related to poor prognosis.

Previous studies examining return to sports after HTO have shown the return to sports rates ranging from 77.3% to 92.3% [3, 8, 19, 24, 31]. Although relatively high return rates have been reported, those previous studies have shortcomings in the study design that impairs accurate assessment of the efficacy of the surgery in functional recovery after HTO. First of all, most of the previous studies included patients who participated in lower impact sports activities such as hiking and cycling, and the averaged activity level on the Tegner activity scale was generally <4. There is little published literature that specifically tracks return to sports activities of higher activity level after HTO. The rates of return to high activity level sports reported in those studies ranged from 75.3% to 100% [8, 13, 18, 30]. The present study demonstrated that approximately 80% of patients who had unilateral HTO returned to high‐impact sports at the presymptomatic level [13]. In the past, we conducted a study examining the return to high‐impact sports after OWHTO and reported a return rate of 75.3%; [13] however, that study included bilateral HTO cases and thus influence of isolated HTO procedure on functional recovery could not be accurately assessed. The present study is designed to include a single surgeon's series with unilateral procedure, enabling accurate assessment of return rate and time to return to the presymptomatic sports activity level. Another unique feature of the present study is the analysis of factors influencing the postoperative functional recovery. Katagiri et al. investigated the risk factors affecting return to sports after OWHTO and showed that the preoperative Tegner score was higher in patients who failed to return to the sports activity at the same level as before surgery; however, their study included the patients with preoperative Tegner activity level ≥2. The results of this study have indicated some factors influencing the poor outcome in return to high‐impact sports [14].

First, postoperative mTFA >3° was indicated as a significant risk factor for poor prognosis. Optimal correction angle in HTO is controversial and still to be determined. Conventionally, postoperative coronal alignment was aimed at slight overcorrection to unload the affected osteoarthritic compartment with the postoperative WBL ratio is usually set to the Fujisawa point, which represents approximately 62% of the width of the tibial plateau; however, recent studies have posed a question to application of the Fujisawa point to all varus KOA, and the ESSKA consensus proposed an individual approach with a WBL ratio of 50%–65% depending on the patient's clinical characteristics [5, 10, 11]. According to Saragaglia et al. [25] a neutral axis knee is the best alignment in consideration of playing sports including running (tennis, jogging and football). Han et al. reported that postoperative mTFA was shown to be negatively associated with overall satisfaction, that is, patients were less satisfied as the amount of overcorrection increased [9]. Postoperative nonphysiological overcorrection may interfere with recovery of the ground support ability and proprioception resulting in unsatisfactory functional recovery. In case of high‐level athletes, therefore, it is imperative to explain the concept of individualized correction to patients at the time of preoperative consultation.

Second risk factor identified in this study was opening gap of more than 10 mm. It is speculated that severe varus knee or advanced KOA requires large amount of correction [23]. It is well known that large correction after OWHTO induced patellofemoral osteoarthritis [16], which is associated with patients' dissatisfaction [22, 28]. In addition, a large correction may induce excessive joint‐line obliquity, which is another factor related to poor prognosis [21].

KL grade 4 was also indicated as a significant risk factor affecting the functional recovery. In the present study, the rate of KL grade 4 was 1 of the 50 knees (2.0%) in the group attaining satisfactory return to presymptomatic sports and four of the 13 knees (30.8%) in the group with failed return to sports. Our previous clinical study for patients undergoing knee osteotomy showed inverse relationship between clinical score and degree of progression of KOA [20]. Therefore, our present and previous studies have indicated that HTO at early‐stage KOA would be recommended for the patients who participate in high‐impact sports.

The strength of this study is the analysis of the study subjects with similar clinical features who desire to return to sports at high activity level. In accordance with evolutions of surgical technique and instruments as well as changes in surgical concept in recent years, the aim and indication of knee osteotomy for KOA patients may have to be modified along the flow of the times. The data obtained in the present study help improve the quality of preoperative decision‐making and consultation, since many patients wish to know whether and when they can return to high‐impact sports activities. Furthermore, establishing realistic goal of surgery at the preoperative stage may prevent both over‐ and underexpectation regarding the surgical efficacy in accomplishment of postoperative return to sports.

Our present study had several limitations to note. First, it was a retrospective study for a specific cohort of patients who opted to receive knee osteotomy with strong motivation for sport activities. Therefore, there is a risk for bias in selection of the study population. Second, the sample size is relatively small, and the follow‐up period is short and varied among the patients (the minimum follow‐up was 24 months with the maximum follow‐up of 102 months). Thus, it remains unclear whether the continued sports activity at the high level may induce recurrent symptoms or osteoarthritic progression during the subsequent follow‐up period. Third, two surgical procedures, OWHTO and OWDTO, with different surgical technique and postoperative rehabilitation protocol were included as surgical interventions. Fourth, presymptomatic sports activity level was determined relying on the patient's recall, which may have induced a recall bias. Fifth, most of the patients had KOA also on the contralateral side, and symptom and function of the contralateral knee may have influenced the postoperative functional recovery. Sixth, muscle strength was not measured in this study. Finally, all the radiological measurements were performed by one observer, though high intra‐ and interrater reliabilities with the use of the software utilized in this study have been confirmed in a previous study [26].

## CONCLUSIONS

Presence of postoperative mTFA > 3° valgus, opening gap >10 mm and KL classification grade 4 were identified as risk factors impairing postoperative return to high‐impact sports.

## AUTHOR CONTRIBUTIONS

All authors were involved in the planning of the study design and in the execution of the study. Hiroshi Nakayama and Shinichi Yoshiya handled the analysis of this study and drafted the manuscript. All authors have corrected and approved the final version of the manuscript.

## CONFLICT OF INTEREST STATEMENT

The authors declare no conflict of interest.

## ETHICS STATEMENT

Ethical approval was granted by Hyogo Medical University (No. 2018). Informed consent is granted by each included patient.

## Data Availability

Production data are available upon reasonable request.
